# Supplementary material for: Treatment Outcomes of Proton Beam Therapy for Esophageal Squamous Cell Carcinoma at a Single Institute
Source: Cancers (Basel). 2023 Nov 22;15(23):5524. doi: 10.3390/cancers15235524 (PMC10705605; doi:10.3390/cancers15235524)
Supplement: Supplementary file 1 [file cancers-15-05524-s001.zip › Table S1.pdf]

Supplementary Table S1. Univariate analysis for overall survival and progression free survival

			Overall survival			Progression free survival		
Factor		Patient (%)	Mean (months)	95% CI (months)	*p*-value	Mean (months)	95% CI (months)	*p*-value
Age	<70	63 (47.7)	63.1	(55.413 – 70.841)	0.070	-	-	0.763
	≥70	69 (52.3)	56.7	(47.692 – 65.803)		-	-	
Gender	Male	121 (91.7)	62.8	(56.270 – 69.402)	0.439	-	-	0.838
	Female	11 (8.3)	40.2	(30.292 – 50.188)		-	-	
ECOG PS	0	62 (47.0)	62.9	(55.805 – 70.025)	0.008	-	-	0.018
	1	65 (49.2)	59.8	(51.272 – 68.389)		-	-	
	2	5 (3.8)	23.0	(2.306 – 43.614)		-	-	
Tumor location	Upper thoracic	16 (12.1)	-	-	0.730	-	-	0.072
	Middle thoracic	53 (40.2)	-	-		-	-	
	Lower thoracic	59 (44.7)	-	-		-	-	
	EG Junction	4 (3.0)	-	-		-	-	
cT classification	T1a	16 (12.1)	46.0	(43.918 – 48.064)	0.000	42.689	(38.045 – 47.333)	0.003
	T1b	75 (56.8)	60.8	(53.386 – 68.301)		59.196	(51.735 – 66.656)	
	T2	18 (13.6)	61.8	(46.606 – 76.898)		63.082	(46.936 – 79.228)	
	T3	23 (17.4)	41.4	(28.486 – 54.359)		29.114	(19.257 – 38.972)	
cN classification	N0	95 (72.0)	67.8	(60.830 – 74.769)	0.000	62.478	(55.364 – 69.592)	0.011

	N1	29 (22.0)	51.8	(39.811 – 63.795)		55.907	(43.140 – 68.673)	
	N2	8 (6.0)	26.4	(12.963 – 39.787)		11.986	(5.770 – 66.246)	
RT dose	< 6600	53 (40.2)	45.2	(37.964 – 52.342)	0.057	49.268	(42.060 – 56.476)	0.537
	≥ 6600	79 (59.8)	66.2	(58.767 – 73.716)		58.139	(50.201 – 66.077)	
PBT technique	Passive scattering	76 (57.6)	58.1	(50.335 – 65.795)	0.081	-	-	0.080
	Pencil beam scanning	56 (42.4)	60.6	(54.303 – 66.811)		-	-	
Concurrent chemotherapy	No	81 (61.4)	65.3	(56.655 – 73.959)	0.067	-	-	0.142
	Yes	51 (38.6)	52.6	(42.988 – 62.140)		-	-	
Concurrent chemotherapy (Stage I)	No	69	65.1	(57.484 – 72.781)	0.251	-	-	
	Yes	22	50.7	(38.614 – 69.538)		-	-	

ECOG PS, European Cooperative Oncology Group Performance score; EG Junction, Esophagogastric junction; RT, Radiotherapy; PBT, Proton beam therapy; CI, Confidence interval
