# Supplementary material for: Treatment Outcomes of Proton Beam Therapy for Esophageal Squamous Cell Carcinoma at a Single Institute
Source: Cancers (Basel). 2023 Nov 22;15(23):5524. doi: 10.3390/cancers15235524 (PMC10705605; doi:10.3390/cancers15235524)
Supplement: Supplementary file 1 [file cancers-15-05524-s001.zip › Table S2.pdf]

Supplementary Table S2. Multivariate analysis for overall survival

		No. of patients (%)	HR	95% CI	*p*-value
cN classification	N0	95 (72.0)	1		
	N1	29 (22.0)	1.782	(0.850 – 3.738)	0.126
	N2	8 (6.0)	5.442	(2.167 – 13.664)	0.000

HR, Hazard ratio; CI, Confidence interval
